# Radionécrose cérébrale chez des patients irradiés pour cancers du nasopharynx: à propos de 3 cas

**DOI:** 10.11604/pamj.2014.19.111.5361

**Published:** 2014-09-30

**Authors:** Abderrahman El Mazghi, Issam Lalya, Kaoutar Loukili, Hanan El Kacemi, Taieb Kebdani, Khalid Hassouni

**Affiliations:** 1 Faculté de Médecine et de Pharmacie, Université Sidi Mohammed Ben Abdallâh et Service de Radiothérapie, CHU Hassan II, Fès, Maroc; 2 Service de Radiothérapie, HIM Mohamed V, Rabat, Maroc; 3 Service de Radiothérapie, Institut National d'Oncologie, Rabat, Maroc

**Keywords:** Radionécrose cérébrale, Cancers du nasopharynx, IRM spectroscopique, Cerebral radiation necrosis, Nasopharyngeal cancer, spectroscopic MRI

## Abstract

La radionécrose cérébrale est une complication tardive, iatrogène, relativement rare de la radiothérapie qui survient après plus de six mois suivant le début du traitement. Elle pourrait s'expliquer par la conjonction de lésions vasculaires, gliales et d'ordre immunologiques. Elle peut mettre en jeu le pronostic fonctionnel et vital du malade. La prévention de cette affection redoutable est fondamentale vu l'absence de traitement potentiellement efficace. Nous rapportons 03 nouveaux cas, chez des patients traités par chimiothérapie d'induction puis radio- chimiothérapie concomitante pour des cancers localement avancés du nasopharynx. Le diagnostic a été orienté par l'IRM spectroscopique et l’évolution était favorable sous corticothérapie dans les 03 cas.

## Introduction

L'incidence des cancers du nasopharynx est élevée au Maroc et aux pays du Maghreb. La radiothérapie est considérée comme le traitement de référence dans la prise en charge des cancers du nasopharynx. Cependant, il n'est pas sans morbidité et les complications peuvent se développer à la suite de dommages aux structures voisines. Le champ de rayonnement couvre inévitablement la région médiane et inférieure des lobes temporaux du cerveau, en raison de la proximité de la base du crâne. En plus, la dose de rayonnement est habituellement de 65 à 70 Gy, qui excède la tolérance du tissu cérébral [1]. Le premier cas de radionécrose cérébrale a été décrit par Fischer et Holfelder en 1930 [2]. Les cas de radionécrose cérébrale rapportés sont encore rares au Maroc. A l'occasion de ces 03 observations, nous rappelons les aspects diagnostiques, thérapeutiques et évolutifs de cette complication avec une revue de la littérature.

## Patient et observation

### Cas 1

**Figure 1:**
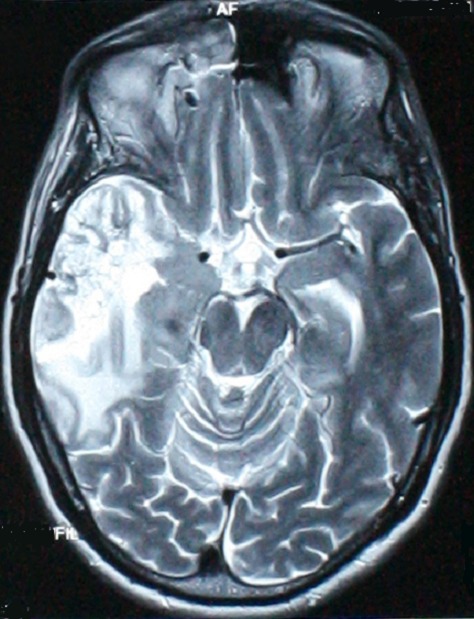
IRM montrant un processus lésionnel temporal en hypo-signal T1, hyper-signal T2, se rehaussant après injection du produit du contraste

**Figure 2:**
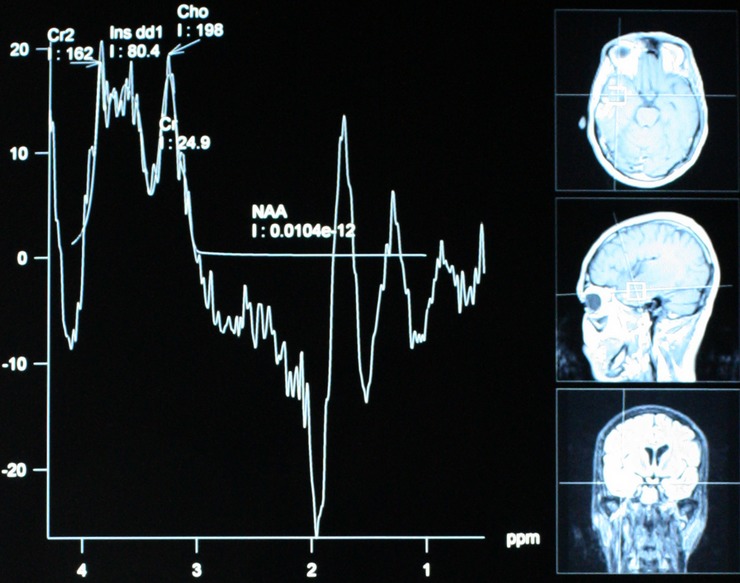
Spectroscopie montrant une légère diminution du NAA avec augmentation de la choline et traces de lactates en faveur d'une radionécrose

Il s'agit d'un patient âgé de 46 ans, suivi depuis 2006 pour carcinome indifférencié type UCNT du nasopharynx, classé initialement T4N1M0, étant donné l'extension à la fosse infra- temporale et au sinus caverneux gauches, il a bénéficié d'une chimiothérapie d'induction suivie d'une radio-chimiothérapie concomitante, à la dose totale de 70 Gy en 35 séances de 2 GY sur 7 semaines, associé à une chimiothérapie hebdomadaire à base des sels du platine à la dose de 30mg/m2 avec une bonne réponse clinique et radiologique. À 33 mois de suivi il s est présenté en consultation avec des céphalées atroces rebelles aux antalgiques usuels avec une désorientation temporo-spatiale. Une TDM cérébrale a mis en évidence une plage hypo-dense temporale gauche compatible avec des localisations secondaires, mais la spectro-IRM redressa le diagnostic en faveur d'une radionécrose (diminution du N-Acétyl-Aspartase (NAA), augmentation de la choline, présence de traces de lactates) (Figure 1, Figure 2). L’évolution était spectaculaire sous corticothérapie.

### Cas 2

**Figure 3:**
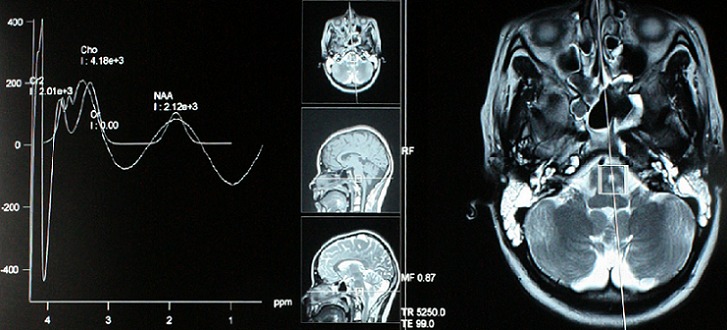
IRM couplée à la spectroscopie montrant un petit foyer lésionnel du bulbe latéralisé à gauche en faveur d'une radionécrose

Il s'agit d'une patiente âgée de 23 ans, suivie depuis 2008 pour carcinome indifférencié type UCNT du nasopharynx, classé T4N2M0 étant donné l'extension à la fosse infra temporale gauche et à la base du crâne, elle a bénéficié d'une chimiothérapie d'induction suivie d'une radio-chimiothérapie concomitante à la dose totale de 70 Gy en 35 séances de 2 GY sur 7 semaines, associé à une chimiothérapie hebdomadaire à base des sels du platine à la dose de 30mg/m2 avec une bonne réponse clinique et radiologique, à 19 mois de suivi elle a présenté une mono-parésie du membre supérieur droit avec une diplopie et un strabisme convergent de l’œil gauche (syndrome alterne du tronc cérébral), avec à l'IRM cérébrale une petit foyer lésionnel du bulbe latéralisé à gauche s’étendant à la jonction bulbo-protubérantielle dont l’étude spectroscopique était en faveur d'une radionécrose d'origine vasculaire (Figure 3). L’évolution était favorable sous corticothérapie, la patiente est toujours en vie avec un bon contrôle locorégional.

### Cas 3

**Figure 4:**
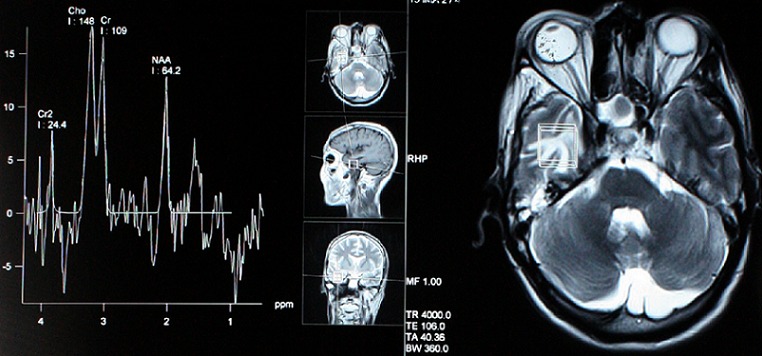
IRM couplée à la spectroscopie montrant un processus lésionnel de la fosse infra-temporale droite et une légère diminution du NAA avec augmentation de la choline en faveur d'une radionécrose

IL s'agit d'un patient âgé de 43 ans, suivi depuis 2007 pour carcinome indifférencié type UCNT du nasopharynx, classé T4N0M0 étant donné l'extension à la fosse infra temporale et au sinus caverneux gauches, il a bénéficié d'une chimiothérapie d'induction suivie d'une radio-chimiothérapie concomitante à la dose totale de 70 Gy en 35 séances de 2 GY sur 7 semaines, associé à une chimiothérapie hebdomadaire à base des sels du platine à la dose de 30mg/m2, avec une bonne réponse clinique et radiologique. À 28 mois de suivi, il s est présenté avec des céphalées, une TDM de contrôle a mis en évidence une plage hypo-dense temporale gauche compatible avec des localisations secondaires, mais la spectro-IRM redressa le diagnostic en faveur d'une radionécrose (diminution du N-Acétyl-Aspartase, augmentation de la choline, présence de traces de lactates) (Figure 4). Le patient était mis sous corticothérapie avec une bonne évolution clinique.

## Discussion

La radionécrose cérébrale, principalement temporale, est une complication bien connue et l'un des plus importants facteurs limitant de la dose dans les cancers du nasopharynx. Son taux d'incidence varie entre 0,95 et 14% selon les séries [1, 3]. Elle pourrait s'expliquer par la conjonction de lésions vasculaires, gliales et d'ordre immunologiques. Les facteurs de risque qui jouent un rôle capital dans le développement d'une radionécrose cérébrale sont l’âge du patient, la chimiothérapie concomitante, la dose totale, la durée de l'irradiation et surtout la dose par fraction avec un rôle protecteur des schémas fractionnés [1, 4]. Les symptômes cliniques de la radionécrose cérébrale sont variables. Elle peut se manifester par des symptômes majeurs, tels que les troubles de conscience et des convulsions ou des plaintes mineures, telles que les vertiges ou les troubles de la mémoire, ou de découverte fortuite lors des examens d imagerie de suivi chez des patients asymptomatiques [1, 4].

L'IRM est l'examen de choix pour le suivi des patients après radiothérapie. Cependant, devant des prises de contraste suspectes, le diagnostic différentiel entre récidive sous forme de métastase cérébrale très exceptionnelle et radionécrose est souvent difficile. La décision diagnostique est importante car en résultent des attitudes thérapeutiques radicalement différentes (surveillance, traitement systémique, chirurgie). La biopsie stéréotaxique est l'une des procédures qui permet d'apporter du matériel anatomopathologique et, par conséquent, un diagnostic fiable mais reste un acte invasif. Les techniques d'IRM multimodales avec les séquences de perfusion et la spectrométrie améliorent grandement la sensibilité et la spécificité de l'examen [4, 5]. Nos malades ont eu une imagerie par résonance magnétique couplée à la spectrométrie et qui a été en faveur de la radionécrose.

L'apport de la TEP au FDG a été évalué dans le suivi des métastases cérébrales irradiées. Si celle-ci est moins sensible et moins spécifique que les dernières techniques IRM citées, elle permet, couplée à l'IRM, d'apporter un argument supplémentaire en faveur de la récidive tumorale si la lésion est hypermétabolique. Sa principale limite est l'hypermétabolisme glucidique cérébral physiologique [5]. La TEP à la 18F-DOPA qui un acide aminé marqué apporte un complément pertinent à l'IRM multimodalité lors du diagnostic différentiel entre radionécrose ou récidive tumorale cérébrale [6]. Le principal objectif du traitement de la radionécrose cérébrale est la guérison clinique avec un risque minimum de morbidité. La résection chirurgicale de la nécrose est hasardeuse et surtout réalisée en cas de syndrome d'hypertension intracrânienne ou de progression rapide des signes cliniques sous traitement symptomatique. De ce fait, l'utilisation des corticoïdes et des traitements symptomatiques est bien souvent le seul recours. La réponse favorable à la corticothérapie est expliquée par l’œdème vasogénique souvent associé à la radionécrose. Il faut cependant souligner la possibilité et les risques des effets secondaires et surtout d'une corticodépendance prolongée. Le traitement par oxygène hyperbare est une alternative qui a été utilisée [1, 4]. Le bévacizumab est un anticorps monoclonal permettant une diminution de la néoangiogénèse et une diminution de la perméabilité de la barrière hémato-encéphalique il est récemment proposé dans le traitement des radionécroses cérébrales réfractaires aux traitements médicamenteux et à l'oxygénothérapie hyperbare [7] Pour le pronostic, Lee a trouvé un taux de survie à 5 ans pour la radionécrose cérébrale de 59%, avec ou sans traitement [8].

## Conclusion

La radionécrose cérébrale est une complication redoutable vue les difficultés diagnostiques et thérapeutiques que pose aux radiothérapeutes. L'IRM classique, couplée aux séquences spectroscopique, a grandement contribué, dans le suivi des patients irradiés, à affiner le diagnostic différentiel entre la récidive tumorale et radionécrose, qui reste cependant difficile. L'obtention d'une preuve histologique par exérèse chirurgicale ou par biopsie stéréotaxique reste à discuter. En absence d'un traitement efficace, la prévention par une meilleure planification dosimétrique est fondamentale.
